# AGC: compact representation of assembled genomes with fast queries and updates

**DOI:** 10.1093/bioinformatics/btad097

**Published:** 2023-03-02

**Authors:** Sebastian Deorowicz, Agnieszka Danek, Heng Li

**Affiliations:** Department of Algorithmics and Software, Faculty of Automatic Control, Electronics and Computer Science, Silesian University of Technology, Akademicka 16, Gliwice 44-100, Poland; Department of Algorithmics and Software, Faculty of Automatic Control, Electronics and Computer Science, Silesian University of Technology, Akademicka 16, Gliwice 44-100, Poland; Department of Data Sciences, Dana-Farber Cancer Institute, Boston, MA 02215, USA; Department of Biomedical Informatics, Harvard Medical School, Boston, MA 02115, USA

## Abstract

**Motivation:**

High-quality sequence assembly is the ultimate representation of complete genetic information of an individual. Several ongoing pangenome projects are producing collections of high-quality assemblies of various species. Each project has already generated assemblies of hundreds of gigabytes on disk, greatly impeding the distribution of and access to such rich datasets.

**Results:**

Here, we show how to reduce the size of the sequenced genomes by 2–3 orders of magnitude. Our tool compresses the genomes significantly better than the existing programs and is much faster. Moreover, its unique feature is the ability to access any contig (or its part) in a fraction of a second and easily append new samples to the compressed collections. Thanks to this, AGC could be useful not only for backup or transfer purposes but also for routine analysis of pangenome sequences in common pipelines. With the rapidly reduced cost and improved accuracy of sequencing technologies, we anticipate more comprehensive pangenome projects with much larger sample sizes. AGC is likely to become a foundation tool to store, distribute and access pangenome data.

**Availability and implementation:**

The source code of AGC is available at https://github.com/refresh-bio/agc. The package can be installed via Bioconda at https://anaconda.org/bioconda/agc.

**Supplementary information:**

Supplementary data are available at *Bioinformatics* online.

## 1 Introduction

Rapidly evolving long-read sequencing technologies such as Pacific Biosciences and Oxford Nanopore have enabled routine haplotype-resolved assembly of haploid and diploid genomes (Cheng *et al.*, 2021; Nurk *et al.*, 2022). We have started to sequence and de novo assemble collections of samples from the same species (Bayer *et al.*, 2020; Ebert *et al.*, 2021; Jayakodi *et al.*, 2020; Leger *et al.*, 2022; Miga and Wang, 2021). For example, the Human Pangenome Reference Consortium (HPRC) has released 94 haplotype assemblies and plans to produce additional 600 assemblies in the next few years (Miga and Wang, 2021; Wang *et al.*, 2022). Nowadays, their total size is about 290 GB of uncompressed FASTA files and in the future it will grow to about 2 TB. These haplotype assemblies do not only encode small variants but also represent complex structural variations in segmental duplications and centromeres, empowering the investigation of genetic sequence variations at full scale for the first time. Currently, we use generic compression tools, such as gzip, to compress collections of similar genomes. Despite high similarity between genomes, these tools can only achieve a 4-fold compression ratio.

There are, however, some specialized tools able to compress better. NAF (Kryukov *et al.*, 2019) is a simple utility that can be used, among others, for FASTA files with genome assemblies. The processing is very simple. Nucleotide symbols are packed 4 into a single byte. Then, they are compressed using a general-purpose zstd compressor. The gains over generic tools are thus little. GeCo3 (Silva *et al.*, 2020) combines neural networks with specific DNA models. This gives better ratios than NAF, but the compression of a 1 GB file takes an hour, making the tool impractical for large collections. HRCM (Yao *et al.*, 2019) implements a specialized variant of the LZSS algorithm (Storer and Szymanski, 1982) that searches for matches between the sequence currently processed and the reference sequence. In addition, it also employs second-level matching, inspired by FRESCO (Wandelt and Leser, 2013) and GDC2 (Deorowicz *et al.*, 2015), in which the same matches are identified between different sequences and the reference for more efficient encoding. The results are compressed using a PPMD compressor (Shkarin, 2002). The compression ratios of HRCM are much better than those of NAF, but even better results are given by the recently published MBGC (Grabowski and Kowalski, 2022) tool. It uses a hash table to index the reference sequence and then looks for matches between the new sequences and the reference. It checks, however, also matches in the reverse-complemented sequence, which sometimes is beneficial. After encoding each contig, MBGC calculates how many symbols are not covered by any match. If this value is larger than about 0.5%, this contig is added to the reference sequences set, i.e. it is indexed using a hash table. Thanks to this approach, parts of the genomes not seen before can serve as references for the remaining genomes of the collections. The matches found are compressed using the LZMA algorithm (Salomon and Motta, 2010).

Both HRCM and MBGC can localize matches among any sequences in the collection. This helps if we aim at the best compression ratio. Nevertheless, the extraction of a selected part of the collection, e.g. a single contig (or sample), is problematic, because we need to decompress everything that was compressed before the contig in query. This takes time, and the time is long if the collection is large. Therefore, these tools are mainly intended to reduce transfer and archival costs. As a result, users have to store uncompressed data for routine analysis. This severely limits their practical applications.

Historically, the first attempts to compress collections of genomes were RLZ (Kuruppu *et al.*, 2010) and GDC (Deorowicz and Grabowski, 2011). Both tools adapted the Ziv–Lempel method to the properties of genome collections. Nevertheless, they assumed that genome collections are given as sets of complete chromosomes and processed the data chromosome by chromosome. Nowadays, the output of *de novo* assembly is just a set of contigs. The contigs are of various sizes, and it is unknown what is the origin of each contig. Thus, the compression problem is much harder and both RLZ and GDC cannot be used here.

In this article, we present AGC (Assembled Genomes Compressor), a highly efficient compression method for the collection of assembled genome sequences of the same species. The compressed collection can be easily extended by new samples. AGC offers fast access to the requested contigs or samples without the need to decompress other sequences. The tool is implemented as a command-line application. Access to the data is also possible using C, C++ and Python programming libraries.

## 2 Materials and methods

### 2.1 The general idea

**Fig. 1.**
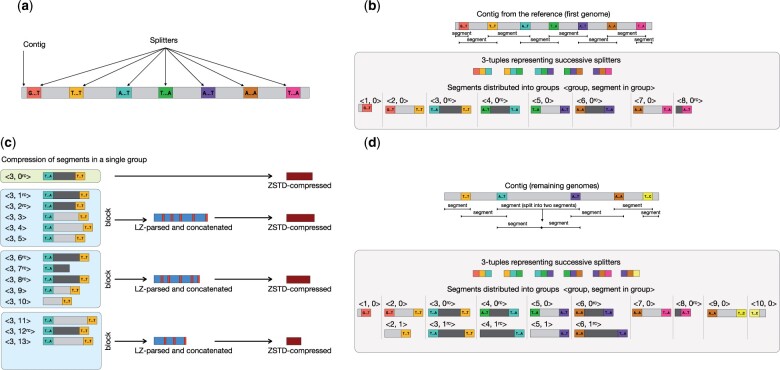
Illustration of the most important stages of the compression algorithm. (**a**) In the first two stages of the compression, we pick some of the *k*-mers of the reference genome (uniformly distributed) as splitters (shown in colors). (**b**) Compression of the first genome. The contigs are split into segments and distributed into groups according to the terminating splitters. For technical reasons some segments are reverse-complemented (dark-gray-marked, ^*rc*^). The 3-tuples represent the successive splitters in the contigs. (**c**) Compression of remaining genomes. The contigs are split into segments and distributed into groups. If there is no group identified by a pair of terminating splitters (e.g. blue and violet) it is checked if there is a triple of consecutive splitters (blue, *any*, violet). If so, it is assumed that the middle splitter (green) is missing (probably due to some evolutionary event) and (blue, violet) segment is split into two segments: each with only one terminating splitter. Otherwise, e.g. for (brown, yellow) segment, a new group is created. (**d**) Compression of segments in a single group. The reference segment is packed using general-purpose zstd compressor. The remaining segments are processed in blocks: LZSS-parsed against the reference segment to find similarities and differences, concatenated (red box is a terminator here) and zstd-compressed

The main contribution of the algorithm is the way it represents the assembled genomes and how it supports fast access to the compressed data. Compression is a three-stage process. Initially, a reference genome (provided by the user) is analyzed to find unique *candidate k*-mers (short sequences of length *k*; 31 by default). In the second stage, the reference is analyzed one more time to find *splitters*, which are candidate *k*-mers distant (in the contigs) from each other by approximately *segment size* (60 kb by default). Proper compression is performed at the third stage, which is executed separately for each added genome. Here, AGC uses splitters to divide each contig into segments. Then, the segments are collected in groups using pairs of terminating splitters to have in the same group segments that are likely highly similar to each other. The actual implementation is more complicated to allow AGC to handle segments with only one terminating splitter (at contig boundaries) or in a situation in which some splitter is ‘missing’ (e.g. due to some evolutionary event). Some insight into such situations is given in Figure 1, and more details are given in the following subsections. The first segment of a group serves as a reference. The remaining segments are processed in blocks (default size 50). Each segment is represented as descriptions of similarities and differences with respect to the reference segment. These (usually short) descriptions are concatenated and compressed with a general-purpose zstd compressor. This allows a highly efficient representation of not only the similarities between segments and the reference segment but also among non-reference segments.

In the decompression of a single contig, it suffices to read the information about the groups and blocks containing the segments of the requested contig. Then, AGC decompresses the reference segments and, partially, also the necessary blocks. In general, the larger the block size, the better the compression ratio, but the longer the access time.

The described strategy is a compromise between compression ratio and access time. It offers very good compression ratios and limits the part of an archive that needs to be decompressed when we want to extract data. This allows us to use AGC archives directly in the routine pipelines, greatly reducing storage costs.

### 2.2 Algorithm overview

The algorithm is implemented as a multithreading application in the C++17 programming language. It supports all IUPAC codes in the input data (A, C, G, T, U, R, Y, S, W, K, M, B, D, H, V, N). The symbols can be lower- and upper-case but before compression sequences are upper-cased. The application can be run in one of the modes:

- *create*—create an archive from FASTA files,
- *append*—add FASTA files to existing archive,
- *getcol*—extract all samples (whole collection) from archive,
- *getset*—extract a sample from archive,
- *getctg*—extract a contig from archive,
- *listset*—list sample names in archive,
- *listctg*—list sample and contig names in archive,
- *info*—show some statistics of the compressed data.

Initially, the application must be run in *create* mode to build a new archive. The user should provide a collection of genomes to compress and a reference genome. The reference genome can be one of the genomes in the collection or a reference for the species. The user can define the parameters of the archive, for example, *k*-mer length (default: 31), *block size* (default: 50) and *segment size* (default: 60 000). Then, the user can extend the archive or ask various types of queries.

There are three main stages of compression:

1. candidate *k*-mer determination,
2. splitters determination and
3. adding genomes to the archive.

The first two occur only in the *create* mode. The last occurs in the *create* and *append* modes.

### 2.3 Candidate *k*-mers determination

In the first stage of compression, all *k*-mers present in the reference contigs are determined and stored in an array. Then, they are sorted using a fast in-place variant of the radix-sort algorithm (Kokot *et al.*, 2018). Finally, the *k*-mers occurring two or more times are removed.

### 2.4 Splitters determination

Each splitter is a candidate *k*-mer. The number of splitters is more or less the reference genome size divided by *segment size*, so for a human genome and default algorithm parameters it is about 50 000. To determine the splitters, AGC processes contigs of a reference genome one by one from the beginning to the end. For each of them, it looks for the first *k*-mer that is a candidate *k*-mer and stores it as a splitter. Then, it skips *segment size* bases and looks for the next splitter, and so on. The last candidate *k*-mer of a contig is also a splitter (cf. Fig. 1a).

### 2.5 Compression of a single genome

The genomes are added to the compressed archive one by one in the same way. The first genome added is, however, the reference genome. The remaining genomes are added in the order provided by the user.

Each genome is compressed contig by contig. At first, each contig is split into *segments*. Segment boundaries are defined by splitters (cf. Fig. 1b). For technical reasons, a splitter is part of both neighbor segments. There are three types of segments:

- *spt-2*—segment surrounded by two splitters; the majority of segments are of this type,
- *spt-1*—segment with only one splitter; this is usually the case at contig boundaries,
- *spt-0*—segment without any splitter; it can happen that for some short or highly redundant contig it is not possible to localize any candidate *k*-mer; such a segment is always a whole contig. Such a situation can also happen if the whole contig contains a sequence not present in the reference genome.

#### 2.5.1 Dealing with *spt-2* segments

Most segments of this type are distributed to groups identified by a pair of splitters they contain. To make this grouping easier, we ‘normalize’ each segment, which means that we compare which of the canonical splitters is lexicographically smaller. Then, we reverse complement the segment if it is necessary to ensure that the smaller splitter is at the beginning of the segment.

The first segment in each group serves as a reference for the group. It is packed: 1, 2, 3 or 4 symbols into a single byte, depending on the alphabet size in the sequence. Then, it is compressed using zstd.

The remaining segments are LZSS-parsed (Storer and Szymanski, 1982) with respect to the reference segment of the group. The (usually) much shorter descriptions of the segments are concatenated into blocks of size *block size*. The blocks are compressed independently using zstd (cf. Fig. 1c and d).

However, it is possible that a segment of this type will be split into two segments of type *spt-1*. This could happen if we have a segment with a pair of splitters (*s*_1_, *s*_2_) and there is no group identified by this pair. In this situation, we check if the archive already contains groups identified by splitter pairs (*s*_1_, *s*_3_) and (*s*_3_, *s*_2_) for any splitter *s*_3_. If so, we LZSS-parse the current segment with respect to the reference segments of both groups to find the best division point. Then we split the segment into two *spt-1* segments. If there is no such pair of groups, the current segment starts a new group (especially this is the case when adding the first genome to the archive).

#### 2.5.2 Dealing with *spt-1* type segments

We analyze all groups in which at least one splitter is the same as the splitter in the current segment. For each such group, we perform LZSS-parsing to find the group in which the cost of storing the current segment will be the smallest. Then, we add the current segment to this group. A segment of this type can also start a new group if the LZSS-parsing shows that there are no groups similar to the current segment.

#### 2.5.3 Dealing with *spt-0* type segments

The segments of type *spt-0* are randomly distributed (but in a deterministic way) into one of 16 groups. Within each group, segments are organized in blocks of size *block size*. Each group is compressed independently using zstd compressor.

### 2.6 Archive organization

The archive contains compressed blocks from all groups. It also contains descriptions of the contigs, i.e. the ids of groups and within-group ids of segments. These descriptions are also zstd-compressed.

### 2.7 Extending an archive

The archive can be extended by new samples. In this mode, the archive is partially loaded into the memory. This means that the last blocks of each group are loaded (but not decompressed until it is necessary to add anything to them). We also load the descriptions of the contigs. Then, we proceed as usual when we add new genomes.

### 2.8 Adaptive mode

For highly divergent species, such as bacteria, better results are possible if the splitters are determined not only in the reference genome but also in the remaining genomes. The processing of the reference is the same as in the default mode. However, when a new genome is added, the *spt-0* type segments are collected in some buffer (not directly stored in the archive). Then, after processing all genome contigs, the *k*-mers in the buffered contigs are counted, duplicates are removed, and also the *k*-mers present in the reference genome are removed to get *sample-candidate k*-mers. Then, these *k*-mers are used to determine new splitters that extend the *global* splitters. After that, the buffered contigs are processed one more time to determine *spt-2*, *spt-1* and *spt-0* segments that are handled as usual.

### 2.9 Decompression of a contig or a sample

To decompress a contig, it is necessary to read its description to find which groups will be required. Then, we zstd decompress the reference segments of the selected groups. If necessary, we also decompress one of the blocks in a group, get LZSS-parsing of the requested segment and reconstruct it. In the query, it is possible to restrict to only some part of a contig. In this case, we decompress only the segments necessary to reconstruct the requested part.

Decompression of a sample is just decompression of the contigs it is composed of.

### 2.10 Block size choice

The block size has a significant impact on the size of the archive, as well as compression and extraction time. We performed a few experiments on the HPRC dataset to measure it. The results will be discussed in the next section. The default *block size* value (50) is a compromise between access time and compression ratio, which should be used for large genomes, such as humans. For bacterial and viral datasets, larger values (e.g. 500) should be a better choice.

## 3 Results

For evaluation, we used several datasets of various species: human, bacterial and viral (Table 1). The machine used in the tests was equipped with an AMD 3990X CPU (64 cores clocked at 2.9 GHz), 256 GiB RAM and 3.6 TB NVME disk. All tools were run with 32 threads. The details of the datasets and the command-lines of the used tools are provided in Supplementary Material.

**Table 1.** Datasets used in the experiment

Dataset	Species	No. of samples	Reference genome size (Mb)	Size (GB)
HPRC	*H.sapiens*	96	3100	293.2
HGSVCp	*H.sapiens*	36	3100	104.4
HGSVCu	*H.sapiens*	36	3100	102.9
SALMO	*S.enterica*	1000	5.03	5.0
CAMP	*C.jejuni*	21 988	1.63	38.5
COVID	*SARS-CoV2*	619 750	0.03	18.8
661K	Bacterial	661 398	90.7	2,640

**Fig. 2.**
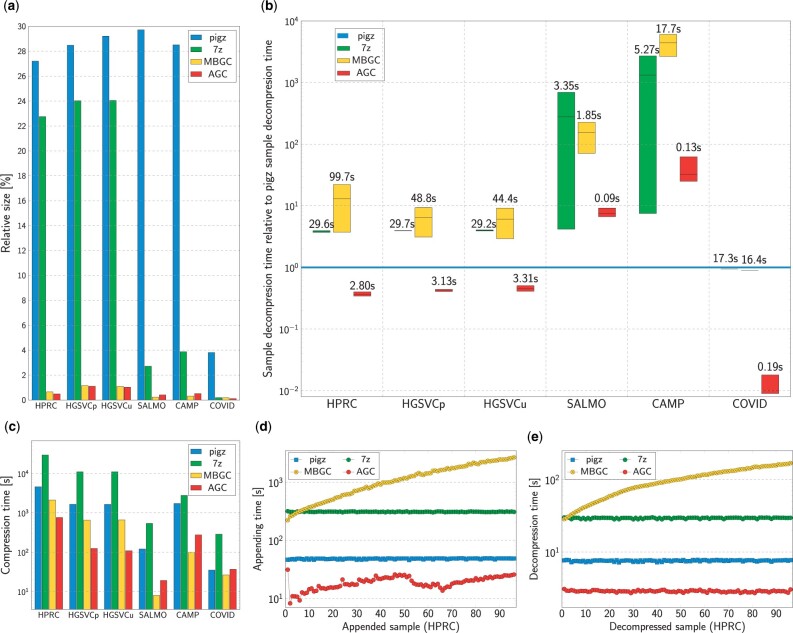
Experimental results. (**a**) Relative sizes of compressed collections of genomes. (**b**) Extraction time of a single sample. The box plots show minimal, median and maximal times relative to decompression time of pigz archives. The labels show median absolute times. (**c**) Compression times of whole collections of genomes. (**d**) Time of appending a single sample to the archive containing given number of HPRC samples (markers). Main memory usage (filled areas). The filled areas of gzip is not presented as they is very small. (d) Appending time of a single HPRC sample to the archive containing given number of samples. (**e**) Extraction time of a single HPRC sample as a function of sample position in the archive

The largest HPRC dataset consists of 94 human haplotype assemblies, reference genome (GRCh38) and CHM13 genome from the T2T consortium (Nurk *et al.*, 2022). With each assembly taking ∼3 GB, the entire dataset requires 293.2 GB of space or 79.8 GB when gzipped. AGC compresses it about 200 times to as little as 1.45 GB in about 12 min using 32 threads. MBGC (Multiple Bacteria Genome Compressor) (Grabowski and Kowalski, 2022) is the only tool that can compete in terms of compression ratio. It produces a 1.95 GB archive in three times longer time (Fig. 2a and c).

The most significant advantage of AGC is the access time to the compressed data allowing one to keep the collection in a compact form and extract the samples, contigs or contig fragments when requested, both from the command line as well as using programming libraries. For example, the complete human sample can be extracted in <3 s, independently of the position of the sample in the collection (Fig. 2b). The contigs can be extracted in even a shorter time. This is tens of times faster than MBGC and even faster than extracting from separate gzip archives (Fig. 2b).

Moreover, in contrast to MBGC, AGC can extend the existing archive with new samples, which takes 10–25 s for a single human genome (Fig. 2d). Extending an MBGC archive means recompressing the whole collection, which takes a lot of time, e.g. a few thousand of seconds to add the last HPRC sample to the archive, and needs a lot of disk space for temporal storage of the decompressed collection. Importantly, AGC needs much less main memory (20 GB) than MBGC (110 GB). The appending time is constant for pigz and 7z as these tools compress each sample separately—they cannot benefit from repetitions in separate genomes for such large genomes.

Figure 2e shows the decompression times of a single sample as a function of the position of the sample in the archive. AGC excels here, decompressing any human sample in <3 s. Moreover, its times are more or less constant. As expected, they are constant also for pigz and 7z. The decompression time for MBGC is much longer than of AGC (about two orders of magnitude for the last samples in the archive). Moreover, the MBGC times increase. This is a consequence of the design of MBGC focused on the best possible ratio and using all of what was seen as a potential reference for what it compresses. Thus, during decompression, MBGC needs to decompress everything that was compressed before the requested genome.

Experiments with two human datasets, phased (HGSVCp) and unphased (HGSVCu), from the Human Genome Structural Variation Consortium, Phase 2 (Ebert *et al.*, 2021) (36 samples each) lead to similar conclusions.

AGC appeared to be quite insensitive to the selection of the reference genome in the human datasets. We examined GRCh38, CHM13 and randomly selected samples as references and differences in compression ratio were always <7% (Table 2).

**Table 2.** Archive sizes for human datasets for reference genome randomly selected from the collection and remaining genomes appended in a random order

Reference sequence	Archive size (MB)
HPRC dataset	
CHM13 draft v1.1	1454
GRCh38	1464
HG00621	1523
HG01071	1473
HG01243	1544
HG02109	1493
HG03486	1496
HGSVCp dataset	
HG00096	1162
HG00731	1150
HG01596	1170
NA19239	1164
NA20847	1179
HGSVCu dataset	
HG00096	1066
HG00731	1101
HG01596	1170
NA19239	1116
NA20847	1063

We also experimented with two bacterial datasets, i.e. SALMO (1000 *Salmonella enterica* samples) and CAMP (21 988 *Campylobacter jejuni* samples) from Blackwell *et al.* (2021). Here, MBGC, a tool designed especially for bacterial genomes, wins in terms of compression ratio by producing smaller archives: 12 MB instead of 21 MB and 121 MB instead of 191 MB. In both cases, the compacted files are rather small, so the difference is not crucial in practice. The reason for the advantage of MBGC is the high diversity of bacterial genomes. Therefore, there is no splitter in some contigs, so the (AGC) grouping of segments does not work as well as for human datasets. To partially overcome the problem, for bacterial data we used an adaptive compression mode, in which AGC extends the list of splitters by *k*-mers from contigs without a known splitter. In addition, we used a larger block size (500) and a shorter segment size (1500).

AGC, on the other hand, clearly leads in extraction times. For a single sample, it needs less than a second, while MBGC needs tens of seconds for the larger dataset (Fig. 2b). Furthermore, the extraction time of AGC increases slowly with increasing collection size. This suggests that even for huge collections, AGC can still be regarded as a way of on-line access to the samples stored in a very compact form.

In the final test in this series, we evaluated the tools for the highly similar collection of ∼620k SARS-CoV2 genomes. AGC wins clearly in terms of compression ratio and access time.

**Fig. 3.**
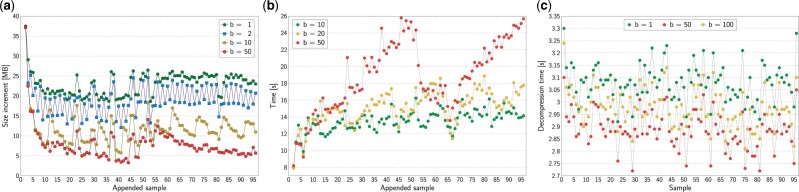
Experimental results. (**a**) Archive space increase after extending the collection by adding a single HPRC sample at a time. The series show impact of block size, *b*. (**b**) Time of extending the collection by adding a single HPRC human sample at a time. The series show impact of block size, *b*. (**c**) Decompression time of a single HPRC sample. The series show impact of block size, *b*

We used the HPRC dataset to evaluate various parameters of AGC, as well as to measure what happens for a single added genome. Figure 3a shows the impact of block size. It is easy to see that for *block size* equal 1, which is more or less equivalent to compressing each genome using only a single reference, we need to spend 20–25 MB for a single genome. When the block size increases, the cost of storing a single genome becomes much smaller, like 5–8 MB for *block size* equal 50. However, this is a compromise, as we can see in Figure 3b. The larger the block size, the slower the extension of the archive. Fortunately, the increase in compression time is moderate. Figure 3c shows the impact of *block size* on decompression. We can notice that value 50 is slightly better than 100, but what is interesting is the time for value 1. The decompression is the slowest here. This is related to the fact that the archive size for *block size* equal to 1 (2889 MB) is much larger than for value 50 (1454 MB) and the time to load the data from the disk is longer than the gains from faster decompression.

To evaluate the flexibility of the examined tools, we performed one more experiment. We used the complete collection of 661 398 bacteria genomes from Blackwell *et al.* (2021) of total size of ∼2.6 TB of uncompressed FASTA files, with 105 324 060 contigs in total. This collection is a mix of various species. Some of them are more frequent, some rare. The quality of assemblies of some are poor (c.f. the average number of contigs is 159 while the average genome size is <4 Mb). Thus, the compression is a real challenge. The results are presented in Table 3. The only tools we were able to run were gzip, 7z and AGC as MBGC failed during decompression, and we were not able to verify the validity of the compressed archive. In terms of compression ratio, AGC is a clear winner. The compression and decompression running times were, however, similar. gzip was the fastest in extraction of a single sample as the samples were compressed separately. Fortunately, the sample extraction of both 7z and AGC are still reasonable. As we can see, AGC can be useful also for such demanding dataset. Nevertheless, in practice, dividing such collection into smaller but more homogeneous subsets would be a better choice.

**Table 3.** Experimental results

Tool	**Compressed size (**GB)	Compression (collection)		Decompression (collection)		Sample extraction (averages)	
		Time (s)	Memory (MB)	Time (s)	Memory (MB)	Time (s)	Memory (MB)
pigz	805.3	80 003	33	5377	6	0.008	2.4
7z.	148.5	252 276	15 395	8049	136	7.18	134
AGC	27.6	121 419	180 873	7529	34 256	8.93	6257
MBGC	Error during compression/decompression						

*Note*: Compression and decompression of 661K dataset.

## 4 Conclusions

Here, we introduce AGC, a versatile package to maintain genome collections in a very compact form. It offers a two-order-of-magnitude reduction of data sizes and allows access to the samples or contigs in seconds or fractions of seconds. Moreover, it allows one to extend the compressed collections by adding new samples. Such a combination of features makes AGC a tool in its own category. This opens new opportunities in the field of (rapidly growing) pangenome projects. AGC archives can be used to distribute pangenome data, store them and quickly answer queries. Due to the programming libraries provided for popular languages, AGC can be easily integrated with existing pipelines, allowing them to operate on small files.

## Supplementary Material

btad097_Supplementary_Data

## Data Availability

All datasets used in the experiments are publicly available. Details on how to download them are given in the Supplementary Data.
